# Self-talk: research challenges and opportunities

**DOI:** 10.3389/fpsyg.2023.1210960

**Published:** 2023-07-03

**Authors:** Thomas M. Brinthaupt, Alain Morin

**Affiliations:** ^1^Department of Psychology, Middle Tennessee State University, Murfreesboro, TN, United States; ^2^Department of Psychology, Mount Royal University, Calgary, AB, Canada

**Keywords:** self-talk, internal dialogue, inner speech, research challenges, methodological challenges, measurement challenge

## Abstract

In this review, we discuss major measurement and methodological challenges to studying self-talk. We review the assessment of self-talk frequency, studying self-talk in its natural context, personal pronoun usage within self-talk, experiential sampling methods, and the experimental manipulation of self-talk. We highlight new possible research opportunities and discuss recent advances such as brain imaging studies of self-talk, the use of self-talk by robots, and measurement of self-talk in aphasic patients.

## Introduction

In this paper we synthesize past and current research findings pertaining to the phenomenon of *self-talk*, the activity of talking to oneself out loud or in silence (Brinthaupt et al., 2009). The latter is usually called *inner speech*, which can be defined as “inner language in the absence of overt and audible articulation” (Langland-Hassan, 2021, p. 2). We include in this definition various related constructs such as *internal monologue* (talking to oneself as one person) or *dialogue* (having a back-and-forth conversation with oneself), *private speech*, and *self-statements* (Morin, 2012, 2019). Self-talk has a long history of theoretical and empirical work (see Vygotsky, 1943/1962; Morin, 2009; Gacea, 2019). There is a great deal of work pertaining to the development, cognitive functions, phenomenology, and neurobiology of self-talk (e.g., Sokolov, 1972; Alderson-Day and Fernyhough, 2015). In recent years, the study of self-talk has been steadily progressing: Latinjak et al. (2023) found 559 articles published between 1978 and 2020 that specifically mentioned “self-talk.”

This large body of work allows for the identification of the main functions of self-talk. These include thinking, problem solving, self-regulation, self-reflection, working memory, task switching, language, rehearsal and replay, emotional expression, thinking about others’ mental states, and self-rumination (see Morin and Racy, 2022, Table 9.1). The array of functions served by self-talk, coupled with the finding that it is present in a significant portion of sampled conscious experiences (Heavey and Hurlburt, 2008), makes it clear that self-talk represents a crucial mental activity.

In this review, we discuss some of the major challenges of studying self-talk, including measurement issues and attempts at assessing self-talk frequency, distinguishing self-talk from other common inner experiences, the study of self-talk in its natural context, and the experimental manipulation of self-talk. We also examine research opportunities and recent advances such as the use of thought sampling procedures to assess self-talk, brain imaging studies of different types and formats of self-talk, the use of self-talk by robots to increase trust during human-robot interactions, and measurement of self-talk in aphasic patients.

## Measurement challenges and opportunities

In our chapter on self-talk assessment in sport (Brinthaupt and Morin, 2020; see Table 3.2), we discuss the main advantages and limitations of most existing self-talk measures (also see Morin and Racy, 2022, Table 9.2). Commonly used self-talk measures include (1) self-report inventories such as the Self-Talk Scale (STS; Brinthaupt et al., 2009) and the Varieties of Inner Speech Questionnaire—Revised (VISQ-R; Alderson-Day et al., 2018); Descriptive Experiential Sampling (DES) (e.g., Heavey and Hurlburt, 2008); recordings of brain activity (e.g., Kühn et al., 2014); think aloud (e.g., Klopp et al., 2020) and thought listing (e.g., Morin et al., 2018) protocols; and observer recordings of self-talk manifestations (e.g., Sokolov, 1972; Van Raalte et al., 1994; Winsler, 2009). Less traditional approaches include videotape reconstruction (e.g., Asendorpf, 1987), one-on-one interviews (e.g., Latinjak et al., 2019), and various experimental methods designed to interfere with self-talk or show self-talk deficits (e.g., Holland and Low, 2010; Tullett and Inzlicht, 2010; Langland-Hassan et al., 2015).

Each method has its strengths and weaknesses. For example, thought listing, interviews, and self-talk recordings are best suited for assessing self-talk content, whereas observer reports and recordings of brain activity tap into the frequency/occurrence of self-talk. The DES approach, recordings of ongoing private speech, and videotape reconstruction offer greater ecological validity compared to interviews or self-report questionnaires. Methods that interfere experimentally with the self-talk process can have more direct control over the nature and content of self-talk compared to thought listing or self-report questionnaires. Self-report methods are easy to use, whereas DES and brain imaging require significantly more time and effort. For more detailed reviews of reliability and validity issues with self-talk measures, see Van Raalte et al. (2019) and Brinthaupt and Morin (2020).

We note two key observations from our review of self-talk measures. First, assessing self-talk *in situ* is difficult—one can adopt methods like private speech recording and DES, but these are either prone to multiple biases (which can affect validity) or are complicated to implement. Second, researchers often must rely on retrospective self-talk descriptions: as soon as a self-report becomes retrospective (even in the short term), it becomes potentially inaccurate because of possible memory biases. There is debate over whether self-report measures inflate actual frequencies of self-talk, as suggested by Hurlburt et al. (2022). Issues with self-report questionnaires, which might possibly bias results pertaining to frequency, include individual differences in interpretating Likert scales (e.g., what does it mean to talk to oneself “rarely” or “often”?) and vagueness or complexity of items (e.g., from the VISQ-R: “When I am talking to myself about things in my mind, it is like I am having a conversation with myself”).

## Methodological challenges and opportunities

There are multiple methodological challenges in self-talk research. Studying the phenomenon in its natural context frequently requires disrupting the flow of the self-talk. For example, self-talk may be “automatic” and outside of our awareness (e.g., Beck, 1976), making it difficult to study *in situ.* Research shows that creating specific self-talk cues or prompts can be effective when learning to meet specific performance goals (e.g., Cutton and Burt, 2023). However, there are potential problems with asking people to recite researcher-determined self-talk and studying its resulting effects. First, such content might not occur naturally in the course of a person’s customary self-talk patterns. Second, the unique nature or style with which people talk to themselves might differ from researcher-provided cue or prompts. In response to these challenges, researchers have developed innovative ways to examine the nature, frequency, and content of self-talk.

One research approach tries to study ongoing (or nearly concurrent) instances of self-talk using experience sampling methods (ESM). Participants typically receive a series of random signals (e.g., via phone or some other device) during their regular daily activities. As soon as possible, they report the content of their inner experiences upon receiving the signal. Researchers have used ESM to validate self-talk measures (e.g., Brinthaupt et al., 2015) and to sample ongoing athletic activity (Dickens et al., 2018). Despite the experience “closeness” of these methods, they still require some degree of interpretation and reporting from the participants that might be susceptible to biases.

A different line of research involves the use of personal pronouns in self-talk. For example, research on “self-distancing” (e.g., Ayduk and Kross, 2010; White et al., 2019) compares the effects of 3rd-person to 1st-person self-talk by asking participants to narrate a personal event with “they/he/she” or with “I/me.” Results show that the increased self-distancing created by 3rd-person self-talk has positive coping effects when people reflect on both past and future negative events. However, it could be argued that this kind of self-talk is unusual and does not typically occur very often naturalistically (i.e., most people typically use first-person “I” in their self-talk; see Bisol, 2021). A related area that has yet to be explored is individual differences in preference for personal pronouns and how these might relate to personality traits.

Another possibility, which has been underutilized in the research literature, is to ask participants to imagine a specific personal or social situation and then report verbatim the kinds of things they would say to themselves as that situation occurs or in response to it having happened. This approach has the potential to provide insight into participants’ typical patterns of self-talk when different kinds of events occur. Some researchers (e.g., Kittani and Brinthaupt, 2023; Łysiak et al., 2023) have asked participants to retrospect about different kinds of prior events (e.g., difficult, negative, or positive) and then report the self-talk and internal dialogues associated with those events. There is also work examining self-talk using prospective or hypothetical situations (e.g., Silk et al., 2020).

Researchers using retrospective or hypothetical approaches must be cognizant of the possibility of biases entering into the self-talk that participants recall or imagine (e.g., Latinjak et al., 2011). Exploring the nature of such potential biases might have important research implications for self-talk processes. For example, when people recall what they may have said to themselves in response to a past situation or event, researchers might examine the extent to which their subsequent thoughts, emotions, and behaviors are likely to be based on what they think happened versus what actually happened.

### Frequency of self-talk

This section is designed to highlight some of the ways that measuring self-talk frequency presents specific research challenges and opportunities. A major challenge in self-talk research has been to quantify the frequency of self-talk (Brinthaupt et al., 2009). A paper by Hurlburt et al. (2022) suggests that, compared to DES frequency results, self-report measures such as the STS over-report actual frequencies of self-talk. We submit that this assertion constitutes an “apples to oranges” comparison fallacy. DES data, if accurate, can only indicate whether volunteers are talking to themselves at specific moments when they are probed, whereas questionnaire data reflect self-talk use in response to specific situations, using subjective frequency scales, and should not be converted to any absolute or relative frequency counts.

Descriptive Experiential Sampling involves a post-data collection interview aimed at double-checking the accuracy of participants’ reports of inner experiences. More fruitful research avenues include examining the effects of a DES interview on subsequent frequency of self-talk reports. Participants might exhibit significant declines in their self-reported self-talk scores after undergoing the DES interview compared to before doing so. That is, they might realize that they talk less often to themselves than they assume.

Self-talk interventions in the sport and clinical domains often rely on the introduction of new or different kinds of self-talk content and studying the effects of that content. For example, an important element of cognitive-behavioral therapy (CBT; Turner et al., 2020; Beck, 2021) is to help people identify their dysfunctional self-talk and then guide them toward replacing those instances with more positive, adaptive, rational, or realistic interpretations of events. There is strong evidence that this approach can be effective with a variety of psychological disorders (e.g., Hofmann et al., 2012). The focus of CBT is on the content of people’s self-talk, rather than with individual differences in the frequency of their everyday self-talk.

One area for future clinical research is to explore whether people who report talking to themselves very often in response to specific situations find it easier to benefit from CBT. Given that CBT aspires to change clients’ negative self-talk (e.g., Luo and McAloon, 2021), it seems logical that individuals who report more frequent self-talk will respond more quickly and favorably to CBT interventions than those who report infrequent self-talk. On the other hand, Van Raalte et al.’s (2016) sport-specific self-talk model predicts that the intentional use of self-talk can deplete a person’s cognitive resources. This suggests that clients whose frequency of intentionally used self-talk is so high that it causes cognitive depletion may be less able to use or benefit from CBT interventions than those with a lower frequency or less cognitively depleting self-talk. It would also be interesting to study how participating in CBT affects people’s awareness of and overall frequency of subsequent self-talk.

### Brain localization of self-talk activity

Early attempts to locate self-talk activity in the brain involved recording neural activity using Positron Emission Tomography (PET) and functional magnetic resonance imaging (fMRI) scans while participants were silently reading single words or sentences, or when they engaged in working memory tasks requiring covert repetition of verbal material (e.g., McGuire et al., 1996; Baciu et al., 1999; Geva et al., 2011). The LIFG within Broca’s area is reliably activated during such simple covert self-talk tasks. Corroborating studies showed that accidental damage to the LIFG, or temporary disruption of LIFG activity using Repetitive Transcranial Magnetic Stimulation (rTMS), leads to self-talk disruption (e.g., Verstichel et al., 1997; Aziz-Zadeh et al., 2005). Other studies looking at in-depth brain activation identified additional brain areas associated with self-talk production, such as Wernicke’s area, the supplementary motor area, insula, left superior parietal lobe, and right posterior cerebellar cortex (e.g., Perrone-Bertolotti et al., 2014).

Of course, self-talk is more than the mere silent reciting of words or sentences. More recent work has examined several variations in self-talk, such as in task-elicited compared to spontaneous self-talk, where the former was linked to decreased activation in Heschl’s gyrus and increased activation in the LIFG, while the latter had the opposite effect in Heschl’s gyrus and no significant effect in LIFG (Hurlburt et al., 2016). Compared to monologic self-talk, dialogic self-talk recruits a broader bilateral group of brain areas, some of which (e.g., right posterior superior temporal gyrus) are also activated when thinking about others’ mental states (Alderson-Day et al., 2016). More recently, Stephan et al. (2020) contrasted inner and overt speech using electroencephalography (EEG) and observed an inhibition of motor areas normally recruited during articulation.

Several research opportunities exist, where a comparison of differential brain activation will likely be noted between different forms of self-talk, including spontaneous, goal-directed, cue-based (instructional), and 1st-person compared to 3rd-person self-talk. To date, researchers have only begun examining how different forms of self-talk are localized in the brain. It is worth noting that while the above line of research is very informative regarding the neural substrates of self-talk, it tells us little about naturally occurring self-talk frequency and content. Recent small and portable ambulatory devices measuring brain activity occurring in natural environments have been developed (see Boto et al., 2018), which most likely will make it possible to identify the brain areas activated during naturally occurring self-talk.

## Research opportunities moving forward

In this last section, we outline a few additional research ideas and current advances pertaining to the phenomenon of self-talk. One opportunity consists in being more creative when using the DES method. For example, Dickens et al. (2018) recorded the actual content of self-talk each time probed participants reported an experience and examined activities participants were engaged in when hearing the beep. One prediction is that complex or challenging activities will generate more self-talk of a problem-solving nature, compared to trivial or repetitive tasks (Brinthaupt, 2019).

Work on self-talk is now permeating Artificial Intelligence research. Pipitone and Chella (2021) and Pipitone et al. (2021) have been trying to enhance human-robot cooperation via self-talk. Humans are exposed to a robot’s self-talk (i.e., human-like self-talk that is programmed into robots) during human-robot interactions. As a result of this exposure, humans are presumed to perceive the robot’s internal processes, and this is thought to increase transparency, trust, and cooperation. Preliminary results are encouraging.

Another fertile research area consists in the study of covert self-talk (inner speech) in aphasics—patients suffering from various language deficits following brain insult. Research on self-talk in aphasics offers interesting new theoretical avenues for brain localization and the relationship between interpersonal and intrapersonal communications. One main question is: do these patients, who exhibit problems with spoken language, experience similar difficulties with covert speech? Although the answer to this question varies depending on which method is used to assess inner speech (Fama and Turkeltaub, 2020), the trend is that covert speech is often preserved (e.g., Fama et al., 2019; Alexander et al., 2023), suggesting that overt and inner speech are clinically dissociable, with the latter being more resistant to brain damage. This research also shows that subjective and objective measures of self-talk are closely related with this population. Stark and colleagues, as well as Fama’s research team, are currently developing self-report measures of self-talk adapted to an aphasic population based on Racy et al. (2019)’s General Inner Speech Questionnaire (B. Stark, personal communication, January 2, 2023; M. Fama, personal communication, January 20, 2023). This emerging research on aphasics illustrates the potential value of studying when self-talk “goes wrong” or is damaged.

## Conclusion

In conclusion, we believe that continued interest in studying the various features of self-talk is warranted. There are many interesting aspects of the phenomenon that have yet to be fully explored. Although there are several methodological and measurement challenges to conducting research on self-talk, recent work offers much promise for additional theoretical developments and new interesting findings.

## Author contributions

Both authors listed have made a substantial, direct, and intellectual contribution to the work, and approved it for publication.

## References

[B1] Alderson-Day B. Fernyhough C. (2015). Inner speech: Development, cognitive functions, phenomenology, and neurobiology. *Psychol. Bull.* 141 931–965. 10.1037/bul0000021 26011789 PMC4538954

[B2] Alderson-Day B. Mitrenga K. Wilkinson S. McCarthy-Jones S. Fernyhough C. (2018). The varieties of inner speech questionnaire—Revised (VISQ-R): Replicating and refining links between inner speech and psychopathology. *Conscious. Cogn.* 65 48–58. 10.1016/j.concog.2018.07.001 30041067 PMC6204885

[B3] Alderson-Day B. Weis S. McCarthy-Jones S. Moseley P. Smailes D. Fernyhough C. (2016). The brain’s conversation with itself: Neural substrates of dialogic inner speech. *Soc. Cogn. Affect. Neurosci.* 11 110–120. 10.1093/scan/nsv094 26197805 PMC4692319

[B4] Alexander J. M. Langland-Hassan P. Stark B. C. (2023). Measuring inner speech objectively and subjectively in aphasia. *Open Sci. Framework.* 10.17605/OSF.IO/8SJNY 37359215

[B5] Asendorpf J. B. (1987). Videotape reconstruction of emotions and cognitions related to shyness. *J. Pers. Soc. Psychol.* 53 542–549. 10.1037//0022-3514.53.3.542 3656085

[B6] Ayduk Ö Kross E. (2010). From a distance: Implications of spontaneous self-distancing for adaptive self-reflection. *J. Pers. Soc. Psychol.* 98 809–829. 10.1037/a0019205 20438226 PMC2881638

[B7] Aziz-Zadeh L. Cattaneo L. Rochat M. Rizzolatti G. (2005). Covert speech arrest induced by rTMS over both motor and nonmotor left hemisphere frontal sites. *J. Cogn. Neurosci.* 17 928–938. 10.1162/0898929054021157 15969910

[B8] Baciu M. V. Rubin C. Décorps M. A. Segebarth C. M. (1999). fMRI assessment of hemispheric language dominance using a simple inner speech paradigm. *NMR Biomed.* 12 293–298. 10484818 10.1002/(sici)1099-1492(199908)12:5<293::aid-nbm573>3.0.co;2-6

[B9] Beck A. T. (1976). *Cognitive therapy and the emotional disorders.* New York, NY: New American Library.

[B10] Beck J. S. (2021). *Cognitive behavior therapy: Basics and beyond*, 3rd Edn. New York, NY: Guilford Publications.

[B11] Bisol S. M. (2021). *How do you talk to yourself? – The effects of pronoun usage and interpersonal qualities of self-talk.* Unpublished Master’s thesis. Available online at: https://scholars.wlu.ca/etd/2364/ (accessed March 12, 2023).

[B12] Boto E. Holmes N. Leggett J. Roberts G. Shah V. Meyer S. S. (2018). Moving magnetoencephalography towards real-world applications with a wearable system. *Nature* 555 657–661. 10.1038/nature26147 29562238 PMC6063354

[B13] Brinthaupt T. M. (2019). Individual differences in self-talk frequency: Social isolation and cognitive disruption. *Front. Psychol. Cogn. Sci.* 10:1088. 10.3389/fpsyg.2019.01088 31156511 PMC6530389

[B14] Brinthaupt T. M. Morin A. (2020). “Assessment methods for organic self-talk,” in *Self-talk in sport*, eds Latinjak A. Hatzigeorgiadis A. (Abingdon: Routledge), 28–50. 10.4324/9780429460623-3

[B15] Brinthaupt T. M. Benson S. A. Kang M. Moore Z. D. (2015). Assessing the accuracy of self-reported self-talk. *Front. Psychol.* 6:570. 10.3389/fpsyg.2015.00570 25999887 PMC4422081

[B16] Brinthaupt T. M. Hein M. B. Kramer T. E. (2009). The Self-Talk Scale: Development, factor analysis, and validation. *J. Pers. Assess.* 91 82–92. 19085287 10.1080/00223890802484498

[B17] Cutton D. M. Burt D. J. (2023). Implementation of self-talk cues for university instructional physical activity programs as a best practice approach. *J. Phys. Educ. Recreation Dance* 94 32–37. 10.1080/07303084.2022.2136309

[B18] Dickens Y. L. Van Raalte J. Hurlburt R. T. (2018). On investigating self-talk: A descriptive experience sampling study of inner experience during golf performance. *Sport Psychol.* 32 66–73. 10.1123/tsp.2016-0073

[B19] Fama M. E. Turkeltaub P. E. (2020). Inner speech in aphasia: Current evidence, clinical implications, and future directions. *Am. J. Speech Lang. Pathol.* 29 560–573. 10.1044/2019_AJSLP-CAC48-18-0212 31518502 PMC7233112

[B20] Fama M. E. Snider S. F. Henderson M. P. Hayward W. Friedman R. B. Turkeltaub P. E. (2019). The subjective experience of inner speech in aphasia is a meaningful reflection of lexical retrieval. *J. Speech Lang. Hear. Res.* 62 106–122. 10.1044/2018_JSLHR-L-18-0222 30950758 PMC6437698

[B21] Gacea A. O. (2019). “Plato and the “internal dialogue”: An ancient answer for a new model of the self,” in *Psychology and ontology in Plato, philosophical studies series*, Vol. 139 eds Pitteloud L. Keeling E. (Cham: Springer International Publishing).

[B22] Geva S. Jones P. S. Crinion J. T. Price C. J. Baron J.-C. Warburton E. A. (2011). The neural correlates of inner speech defined by voxel-based lesion–symptom mapping. *Brain* 134 3071–3082. 10.1093/brain/awr232 21975590 PMC3187541

[B23] Heavey C. L. Hurlburt R. T. (2008). The phenomena of inner experience. *Conscious. Cogn.* 17 798–810. 18258456 10.1016/j.concog.2007.12.006

[B24] Hofmann S. G. Asnaani A. Vonk I. J. Sawyer A. T. Fang A. (2012). The efficacy of cognitive behavioral therapy: A review of meta-analyses. *Cogn. Ther. Res.* 36 427–440. 10.1007/s10608-012-9476-1 PMC3584580 23459093

[B25] Holland L. Low J. (2010). Do children with autism use inner speech and visuospatial resources for the service of executive control? Evidence from suppression in dual tasks. *Br. J. Dev. Psychol.* 28 369–391. 10.1348/026151009X424088 20481393

[B26] Hurlburt R. T. Alderson-Day B. Kühn S. Fernyhough C. (2016). Exploring the ecological validity of thinking on demand: Neural correlates of elicited vs. spontaneously occurring inner speech. *PLoS One* 11:e0147932. 10.1371/journal.pone.0147932 26845028 PMC4741522

[B27] Hurlburt R. T. Heavey C. L. Lapping-Carr L. Krumm A. E. Moynihan S. A. Kaneshiro C. (2022). Measuring the frequency of inner-experience characteristics. *Perspect. Psychol. Sci.* 17 559–571. 34283671 10.1177/1745691621990379

[B28] Kittani S. R. Brinthaupt T. M. (2023). Exploring self-talk in response to disruptive and emotional events. *J. Constr. Psychol.* 10.1080/10720537.2023.2194691

[B29] Klopp E. Schneider J. F. Stark R. (2020). *Thinking aloud: The mind in action.* Weimar: Bertuch Verlag GmbH.

[B30] Kühn S. Fernyhough C. Alderson-Day B. Hurlburt R. T. (2014). Inner experience in the scanner: Can high fidelity apprehensions of inner experience be integrated with fMRI? *Front. Psychol.* 9:1393. 10.3389/fpsyg.2014.01393 25538649 PMC4260673

[B31] Langland-Hassan P. (2021). Inner speech. *WIREs Cogn. Sci.* 12:e1544. 10.1002/wcs.1544 32949083

[B32] Langland-Hassan P. Faries F. R. Richardson M. J. Dietz A. (2015). Inner speech deficits in people with aphasia. *Front. Psychol.* 6:528. 10.3389/fpsyg.2015.00528 25999876 PMC4419662

[B33] Latinjak A. T. Morin A. Brinthaupt T. M. Hardy J. Hatzigeorgiadis A. Kendall P. C. (2023). Self-talk: An interdisciplinary review and transdisciplinary model. *Rev. Gen. Psychol.*

[B34] Latinjak A. T. Torregrosa M. Renom J. (2011). Combining self-talk and performance feedback: Their effectiveness with adult tennis players. *Sport Psychol.* 25 18–31. 10.1123/tsp.25.1.18

[B35] Latinjak A. T. Torregrossa M. Comoutos N. Hernando-Gimeno C. Ramis Y. (2019). Goal-directed self-talk used to self-regulate in male basketball competitions. *J. Sports Sci.* 37 1429–1433. 10.1080/02640414.2018.1561967 30616448

[B36] Luo A. McAloon J. (2021). Potential mechanisms of change in cognitive behavioral therapy for childhood anxiety: A meta-analysis. *Depress. Anxiety* 38 220–232. 10.1002/da.23116 33225527

[B37] Łysiak M. Puchalska-Wasyl M. M. Jankowski T. (2023). Dialogues between distanced and suffering I-positions: Emotional consequences and self-compassion. *J. Constr. Psychol.* 1–14.

[B38] McGuire P. K. Silbersweig D. A. Wright I. Murray R. M. Frackowiak R. S. J. Frith C. D. (1996). The neural correlates of inner speech and auditory verbal imagery in schizophrenia: Relationship to auditory verbal hallucinations. *Br. J. Psychiatry* 169 148–159. 10.1192/bjp.169.2.148 8871790

[B39] Morin A. (2009). “Inner speech and consciousness,” in *Encyclopedia of consciousness*, ed. Banks W. (Amsterdam: Elsevier).

[B40] Morin A. (2012). “Inner speech,” in *Encyclopedia of human behavior*, 2nd Edn, ed. Hirstein W. (San Diego, CA: Elsevier), 436–443.

[B41] Morin A. (2019). When inner speech and imagined interactions meet. *Imagin. Cogn. Pers.* 39 374–385. 10.1177/0276236619864276

[B42] Morin A. Racy F. (2022). “Frequency, content, and functions of self-reported inner speech in young adults: A synthesis,” in *Inner speech, culture & education*, ed. Fossa P. (Cham: Springer).

[B43] Morin A. Duhnych C. Racy F. (2018). Self-reported inner speech use in university students. *Appl. Cogn. Psychol.* 32 376–382. 10.1002/acp.3404

[B44] Perrone-Bertolotti M. Rapin L. Lachaux J. P. Baciu M. Loevenbruck H. (2014). What is that little voice inside my head? Inner speech phenomenology, its role in cognitive performance, and its relation to self- monitoring. *Behav. Brain Res.* 261 220–239. 10.1016/j.bbr.2013.12.034 24412278

[B45] Pipitone A. Chella A. (2021). What robots want? Hearing the inner voice of a robot. *iScience* 24:102371. 10.1016/j.isci.2021.102371 33997672 PMC8101072

[B46] Pipitone A. Geraci A. D’Amico A. Seidita V. Chella A. (2021). Robot’s inner speech effects on trust and anthropomorphic cues in human-robot cooperation. *arXiv* [preprint]. arXiv:2109.09388.

[B47] Racy F. Morin A. Duhnych C. (2019). Using a thought listing procedure to construct the general inner speech questionnaire: An ecological approach. *J. Constr. Psychol.* 33 385–405. 10.1080/10720537.2019.1633572

[B48] Silk J. S. Pramana G. Sequeira S. L. Lindhiem O. Kendall P. C. Rosen D. (2020). Using a smartphone app and clinician portal to enhance brief cognitive behavioral therapy for childhood anxiety disorders. *Behav. Ther.* 51 69–84. 10.1016/j.beth.2019.05.002 32005341 PMC6995786

[B49] Sokolov A. N. (1972). *Inner speech and thought.* New York, NY: Plenum Press.

[B50] Stephan F. Saalbach H. Rossi S. (2020). The brain differentially prepares inner and overt speech production: Electrophysiological and vascular evidence. *Brain Sci.* 10:148. 10.3390/brainsci10030148 32143405 PMC7139369

[B51] Tullett A. M. Inzlicht M. (2010). The voice of self-control: Blocking the inner voice increases impulsive responding. *Acta Psychol.* 135 252–256. 10.1016/j.actpsy.2010.07.008 20692639

[B52] Turner M. J. Wood A. G. Barker J. B. Chadha N. (2020). “Rational self-talk: A rational emotive behaviour therapy (REBT) perspective,” in *Self-talk in sport*, eds Latinjak A. Hatzigeorgiadis A. (Abingdon: Routledge), 109–122.

[B53] Van Raalte J. L. Brewer B. W. Rivera P. M. Petitpas A. J. (1994). The relationship between observable self-talk and competitive junior tennis players’ match performances. *J. Sport Exerc. Psychol.* 16 400–415.

[B54] Van Raalte J. L. Vincent A. Brewer B. W. (2016). Self-talk: Review and sport-specific model. *Psychol. Sport Exerc.* 22 139–148. 10.1016/j.psychsport.2015.08.004

[B55] Van Raalte J. L. Vincent A. Dickens Y. L. Brewer B. W. (2019). Toward a common language, categorization, and better assessment in self-talk research: Commentary on “Speaking clearly…10 years on.” *Sport Exerc. Perform. Psychol.* 8 368–378. 10.1037/spy0000172

[B56] Verstichel P. Bourak C. Font V. Crochet G. (1997). Langage intérieur après lésion cérébrale gauche: Etude de la représentation phonologique des mots chez des patients aphasiques et non aphasiques [Inner speech following left hemispheric lesion: A study of the phonological representation of words in aphasic and non-aphasic participants]. *Neuropsychology* 7 281–283.

[B57] Vygotsky L. S. (1943/1962). *Thought and language.* Cambridge, MA: MIT Press.

[B58] White R. E. Kuehn M. M. Duckworth A. L. Kross E. Ayduk Ö (2019). Focusing on the future from afar: Self-distancing from future stressors facilitates adaptive coping. *Emotion* 19 903–916. 10.1037/emo0000491 30221949

[B59] Winsler A. (2009). “Still talking to ourselves after all these years: A review of current research on private speech,” in *Private speech, executive functioning, and the development of verbal self-regulation*, eds Winsler A. Fernyhough C. Montero I. (New York, NY: Cambridge University Press), 3–41.

